# The Significance of Exposure to Pregestational Type 2 Diabetes in Utero on Fetal Renal Size and Subcutaneous Fat Thickness

**DOI:** 10.1155/2022/3573963

**Published:** 2022-06-30

**Authors:** Christy L. Pylypjuk, Chelsea Day, Yasmine ElSalakawy, Gregory J. Reid

**Affiliations:** ^1^Department of Obstetrics, Gynecology and Reproductive Sciences, University of Manitoba, Winnipeg R3A1R9, Canada; ^2^Children's Hospital Research Institute of Manitoba, Winnipeg R3E3P4, Canada

## Abstract

**Objectives:**

To determine the relationship between exposure to pregestational type 2 diabetes (T2D) and renal size and subcutaneous fat thickness in fetuses during routine obstetrical ultrasound.

**Methods:**

This was a case-control study (January 1, 2019 to December 31, 2019). Routine obstetrical ultrasounds performed between 18 and 22 weeks' gestation at a tertiary-care fetal assessment unit were reviewed. “Cases” comprised ultrasounds of fetuses exposed to pregestational T2D in utero. The control group was assembled from ultrasounds of healthy controls. Postprocessing measurements of fetal renal size and abdominal wall thickness from stored images were performed by two independent observers, and findings were compared between groups.

**Results:**

There were 54 cases and 428 ultrasounds of healthy controls. The mean maternal age of cases was 32.1 years (SD 6.2) compared to 33.2 years (SD 5.3) for healthy controls, and the majority of ultrasounds were performed in multiparous patients (83%). At the 18 to 22 week ultrasound, there was a significant reduction in renal size amongst fetuses exposed to maternal T2D in utero compared to controls; among cases, the mean renal width was 8.0 mm (95% CI 7.8–8.1) compared to 11.4 mm (95% CI 10.6–12.7) in controls (*p* < 0.0001); the mean renal thickness among cases was 8.1 mm (95% CI 7.9–8.2) compared to 11.5 mm (95% CI 10.7–12.9) in controls (*p*=0.001). There was no obvious difference in estimated fetal weight between groups, yet fetuses exposed to maternal T2D had increased subcutaneous abdominal wall fat thickness at this early gestational age (*p*=0.008).

**Conclusions:**

Fetal renal size in cases exposed to pregestational T2D is significantly smaller compared to controls, and subcutaneous abdominal wall fat is significantly thicker. Given emerging evidence about the developmental origins of disease, further study is needed to correlate the association between fetal renal size and fat distribution in the fetus and the long-term risk of chronic renal disease and diabetes in these offspring.

## 1. Introduction

Type 2 diabetes (T2D) is increasing globally and along with it, the number of affected pregnancies [1, 2]. Pregestational diabetes is associated with multiple perinatal complications for both mothers and offspring [3–5]. For women with T2D, pregnancy-related risks span from preconception to postpartum. For offspring, these risks begin in utero but also confer a heightened lifelong risk of diabetes, obesity, and other medical conditions [3–9]. While family history of T2D is a known risk factor for developing diabetes, there is emerging evidence that suggests a worsening disease severity with each successive generation affected [10–12]. Additionally, T2D is associated long-term with chronic renal disease including the need for dialysis or transplant [13–15]. The time between diagnosis of T2D and renal failure is tightly correlated. However, with an earlier age of diagnosis, the age of those requiring dialysis is also decreasing [15–17].

One of the challenges of studying the impact of diabetes in pregnancy is that it can be difficult to accurately differentiate cases of “true” gestational diabetes, diagnosed ∼26 to 28 weeks of pregnancy, from those with previously undiagnosed T2D. Pregestational diabetes in reproductive-aged women has traditionally referred to type 1 diabetes, but in our region, T2D is the predominant form of pre-existing diabetes in pregnancy [18]. Our region also experiences high rates of youth-onset T2D and we anticipate trends towards earlier onset of comorbidities of diabetes including end-stage renal failure requiring dialysis with earlier ages at diagnosis of T2D [18, 19]. Risk of youth-onset diabetes is also independently correlated with childhood obesity [13, 20]. It has been shown that renal tubule number is reduced in childhood-onset diabetes [21–23]; however, less is known about the relationship between fetal kidneys size or trajectory of renal volumes into childhood and other markers of diabetic nephropathy in these offspring. There is also a gap in understanding the relationship between fetal renal volumes and postnatal function [22, 23]. Despite the known association between childhood obesity and youth-onset T2D, little attention has been paid to fetal fat distribution beyond overall fetal growth or macrosomia. With increasing awareness of the developmental origins of health and disease (DOHaD), there is speculation as to whether fetal renal injury and/or metabolic changes may already begin in utero for offspring of mothers with diabetes [22, 24–26].

Imaging of the fetal kidneys in a single, transverse plane of the abdomen is a required view during routine obstetrical ultrasound at 18 to 22 weeks' gestation—also known as the “anatomy scan” [27, 28]. This view serves as a primary screen for confirming the presence of both kidneys and for detection of renal pyelectasis or other renal anomalies. While two-dimensional renal size can be evaluated from this image, measurements are not routinely performed nor additional views taken (i.e., sagittal or coronal planes) unless pathology is seen or suspected. The goal of this study was to determine the relationship between in utero exposure to maternal T2D and fetal renal size and subcutaneous abdominal wall fat thickness during routine obstetrical ultrasound. Results of this study can be used to explore intergenerational linkages between maternal T2D and the risk of renal disease and diabetes in offspring, and for developing early prevention strategies using fetal ultrasound markers of chronic disease risk.

## 2. Materials and Methods

This was a historical case-control study conducted at a tertiary hospital fetal assessment unit in Canada over a one-year period (January 1, 2019 to December 31, 2019). This hospital serves as one of two regional referral centers for specialized obstetrical care for a total population of 1.3 million inhabitants and a geographic region which includes urban, rural, and northern/remote communities. The hospital provides care for the highest concentration of cases of complex diabetes in the region (including diabetes in pregnancy), and there are approximately 5,500 deliveries per year; the regional fetal assessment program performs over 10,000 ultrasounds annually and served as the study site. Research ethics approval was obtained from the University of Manitoba Health Research Ethics Board.

All routine obstetrical ultrasounds performed between 18 and 22 weeks' gestation within the fetal assessment unit during the study period were reviewed for eligibility. “Cases” were defined as ultrasounds of singleton fetuses exposed to pregestational maternal T2D in utero. Controls were assembled from ultrasounds of healthy maternal controls (without comorbid conditions) during the same study period. Ultrasounds were excluded if there were other coexisting maternal medical conditions (including type 1 diabetes), congenital anomalies, genetic syndromes, or twins and higher-order multiples. Patients with type 1 diabetes were excluded as not all would have been routinely referred to our fetal assessment unit for their 18 to 22 week anatomy scan, and type 1 diabetes represents the minority of pregestational diabetes cases in our region. Cases were also excluded if there were no stored images of the fetal kidneys or abdomen between 18 and 22 weeks' gestation. Hand-searches of the fetal assessment case lists identified all potentially eligible ultrasounds during the study period. Fetal assessment reports were then evaluated by experienced research personnel to isolate all cases and controls meeting eligibility criteria and collate information regarding basic demographics and relevant medical history using standardized data collection sheets. Lab and physical exam data are not routinely collected in our fetal ultrasound records. Thus, surrogate markers of glycemic control and diabetes management were identified in the clinic notes as recorded by the sonographer at time of scan per patient self-report. Maternal glycemic control was coded as either “normal” (if blood glucose readings were at target and no additional medication had been prescribed) or “poor” if several glucose readings were above target (defined by any of: (i) fasting glucose >5.3 mmol/L; (ii) 1-hour postprandial glucose >7.8 mmol/l; and (iii) 2-hour postprandial glucose >6.7) and/or written in the ultrasound record as “poor control” [29]. Maternal therapy for diabetes was coded as insulin, oral medication, both, or diet alone. Medical comorbidities, including hypertension, were identified from the ultrasound report and/or the prenatal record or referral letter. The residence was coded as urban, rural, or northern by the forward sortation area.

Postprocessing review of stored ultrasound images and reports was performed to obtain data about fetal biometry and measurements of renal size and abdominal wall thickness. Standard two-dimensional measurements of fetal renal width (also known as “anterior-posterior diameter”) and thickness (also known as “transverse diameter”) were performed [30, 31] and abdominal wall thickness measurements were obtained in a standardized fashion using the approach by Higgins et al. [32]. A second observer, blinded to the initial observations and case-status, performed repeat measurements of fetal renal width and thickness and abdominal wall thickness in a random selection of 25% of ultrasounds to insure interobserver reliability. Where multiple ultrasounds were performed between 18 and 22 weeks for the same pregnancy, the earliest ultrasound was chosen and used to obtain the measurements of interest.

Statistical analysis was performed using Stata v.14.2 (Stata Corp LLC, College Station, TX) software. Continuous variables were presented as means with standard deviations if normally distributed or as medians with interquartile ranges if nonparametrically distributed. Dichotomous and categorical variables were described as proportions. Student *t*-test, chi-square test, and Kruskal–Wallis tests were used to compare outcomes between groups depending on data type and distribution. Regression analyses were performed to evaluate the relationships that estimated fetal weight and abdominal circumference had on renal size and abdominal wall thickness. The Spearman correlation coefficient was used to evaluate the interobserver reliability of renal and abdominal wall thickness measurements.

## 3. Results

**Figure 1:**
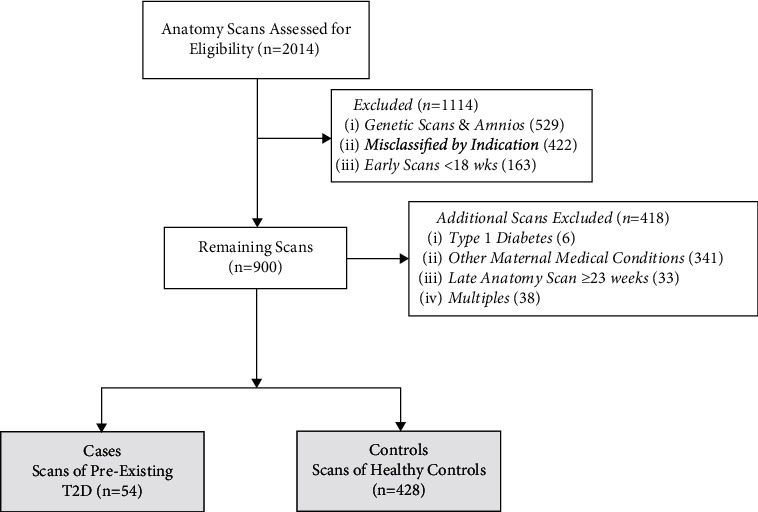
Flow diagram of study subjects. amnio = genetic amniocentesis.

2014 “routine anatomy” ultrasounds were performed in the Fetal Assessment Unit during the study period (Figure 1). Initially, 1114 ultrasounds were excluded because they were performed too early (prior to 18 weeks' gestation), for known congenital anomalies or suspected genetic conditions, or as a follow-up scan to re-evaluate other pregnancy complications unrelated to the primary fetal anatomic survey. Upon further review, another 418 ultrasounds were excluded due to the presence of other comorbid maternal medical conditions (including 6 patients with type 1 diabetes), twins or higher-order multiples, and/or because the referral was for a late anatomy ultrasound beyond 23 weeks and outside of the gestational age range under study. The remaining 482 ultrasounds met inclusion criteria and were included in the case-control analysis (54 cases with fetal exposure to pregestational maternal T2D and 428 controls) (Figure 1). The mean maternal age of cases with T2D was 32.1 years (SD 6.2) compared to 33.2 years (SD 5.3) for healthy controls (*p* = 0.160). 33% of patients resided in rural/northern communities; the remainder resided in urban areas, and there was no difference between cases and controls. The majority of patients with T2D were treated with insulin (85.2%) and 13% were treated with oral medications (primarily metformin); one person was managed with diet alone. 18.5% of patients with T2D were identified as having poor glycemic control. The majority of ultrasounds were performed on multiparous women (83%) and was similar for both groups (*p* = 0.461).

**Figure 2:**
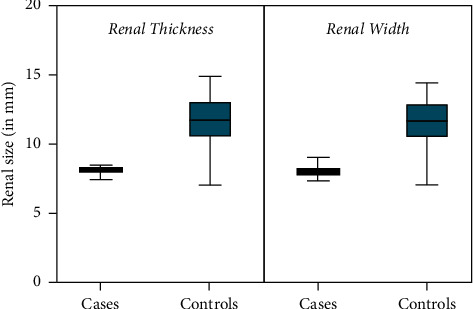
Fetal renal thickness and width (means with 95% confidence intervals) comparing cases and controls.

**Figure 3:**
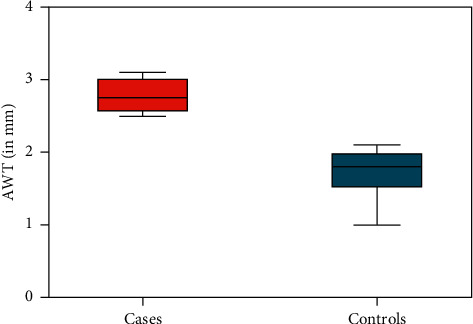
Fetal abdominal wall thickness (means with 95% confidence intervals) comparing cases and controls. AWT = abdominal wall thickness.

**Figure 4:**
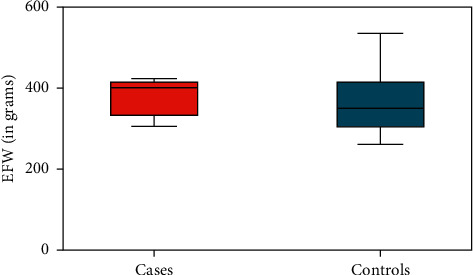
Estimated fetal weight (means with 95% confidence intervals) comparing cases and controls. EFW = estimated fetal weight.

At the time of routine obstetrical ultrasound between 18 and 22 weeks,' fetal renal size was significantly smaller amongst cases compared to controls. The mean renal width in cases exposed to T2D was 8.0 mm (95% CI 7.3–8.7) compared to 11.4 mm (95% CI 10.8–12.7) in controls (*p* < 0.0001); the mean renal thickness among cases was 8.1 mm (95% CI 7.6–8.4) compared to 12.5 mm (95% CI 11.1–13) in controls (*p*=0.001), representing an almost 35% reduction in renal size in each dimension with exposure to maternal T2D in utero (Figure 2). There was no obvious difference in the mean estimated fetal weight at midtrimester between groups (398 grams (95% CI 331–407) in cases compared to 349 grams (95% CI 303–401) in controls (*p*=0.4273), although fetuses exposed to T2D had significantly thicker subcutaneous abdominal wall fat already at this early gestational age (Figures 3 and 4). For cases, the mean abdominal wall thickness was 2.8 mm (95% CI 2.6–3) versus 1.8 mm (95% CI 1.5–1.9) for controls (*p*=0.008). There was a weak correlation between estimated fetal weight and abdominal wall thickness (*r* = 0.151; *p*=0.001) at 18 to 22 weeks' but no obvious relationship between fetal weight and renal size (for renal width, *r* = 0.0003 and *p*=0.945; for renal thickness, *r* = 0.011 and *p*=0.814). There was good interobserver correlation of renal and abdominal wall measurements (*r* = 0.892; *p* < 0.0001).

## 4. Discussion

As we emerge from the COVID-19 pandemic, information about the fetal impact of exposures in utero remains important for tackling another global health crisis-the current diabetes epidemic [1]. With estimations that the global prevalence of T2D is expected to double by the year 2030, the associated risks of renovascular sequelae, end-stage renal disease, and increased demands for dialysis and transplant also looms [1, 14, 18]. Specifically, the incidence of youth-onset T2D is steadily increasing worldwide and is closely linked to rising rates of childhood obesity [18, 19]. However, much of our current understanding of complications of childhood T2D and related end-organ sequelae is informed by knowledge of disease processes in adults and other comorbidities of obesity. There is particular concern that the earlier onset of disease in children could be linked to an earlier age of developing complications of diabetes including diabetic nephropathy [18–20, 33, 34]. Observed trends towards increasing severity of T2D with each successive generation affected have heightened the speculation about whether there may be factors beyond genetics and environment that are mediating this phenomenon [33]. Children of mothers with pregestational T2D are known to have an increased baseline risk of developing diabetes and renal disease later in life, and our study provides some evidence that differences in renal size can already be seen in utero: fetuses exposed to T2D in our population had kidneys that were ∼35% smaller in both dimensions compared to healthy controls. While some changes to renal architecture are known amongst those diagnosed with diabetes in childhood, we were somewhat surprised that a significant difference could already be observed by gross two-dimensional measurements of fetal kidneys by midpregnancy. Renal measurements obtained for our control group are consistent with published reference ranges in the general population [30, 31] and attests to the selection of controls and enhances the generalizability of our results. By simply applying measurement calipers to standardly obtained ultrasound images, the potential use of fetal renal size to enhance risk-stratification of offspring deemed high-risk for developing diabetes and renal disease would not require any extra healthcare dollars nor use of additional resources, but does need further investigation. Given the burden of diabetes and associated nephropathy, any potential opportunities to prevent onset or reduce complications need to be pursued aggressively. Our study needs validation by other longitudinal studies to evaluate the impact of renal size on risk of chronic kidney disease, but if a correlation exists, this would suggest that future prevention strategies may include formal use of fetal ultrasound followed by targeted health-promotion strategies in early childhood [35, 36].

Despite the well-known association between obesity and the risk of diabetes, we were surprised to find that thicker subcutaneous abdominal wall fat was already measureable in utero amongst fetuses exposed to maternal T2D and that this relationship did not appear to be mediated by a difference in overall fetal weight as estimated by ultrasound. We postulate that these findings might reflect a particular predisposition to obesity, particularly central obesity, amongst offspring of mothers with T2D which might already have begun in the womb. Central obesity and abdominal wall fat have already been connected to health complications in children and adults with and without diabetes, but unlike other fetal ultrasound papers that have looked at the impact of gestational diabetes, ours is one of the few studies to describe a possible linkage between fat distribution in utero and prenatal exposure to maternal T2D [37–39]. Antenatally, much of the interest in fetal size for pregnant women with diabetes (namely fetal overgrowth or “macrosomia”) is related to the short-term risks of obstetrical complications including stillbirth, shoulder dystocia, asphyxia, and intrapartum birth injuries, among others [4, 40–42]. However, by focusing exclusively on estimated fetal weight, we may be limiting our understanding of underlying fetal metabolism and lipid profile or other offspring risks which might also relate to health outcomes beyond the neonatal period. Additionally, if thickened subcutaneous abdominal wall fat in the fetus is predictive of future health risk, it could confer the earliest possible opportunity for intervention to combat childhood obesity. One particular risk of retrospective studies evaluating diabetes in pregnancy is related to misclassification bias which exists from the inclusion of cases with gestational diabetes (GDM), which may instead represent a group of women who have yet to be diagnosed T2D and not diabetes of gestational onset. Unless preconceptional diabetes testing was performed a priori, this risk of bias and/or confounding of results exists. One retrospective study published about the role of AWT in predicting the onset of GDM later in pregnancy showed that fetuses of mothers with GDM had thicker AWT at midpregnancy [43]. For our study, the decision to restrict analysis to those patients with confirmed T2D not only eliminates a potential risk of misclassification bias or confounding by GDM, but if the aforementioned study is true, our significant difference in AWT between cases and controls may in fact represent an attenuated result. Because we cannot confirm if any patients in the control group had undiagnosed T2D or went on to develop GDM, there is a chance that the significant difference in subcutaneous fat thickness is actually an under-representation of the true degree of difference between groups and that AWT differences between cases and controls may be even greater than measured in our population. Ongoing work is needed to elucidate any potential linkage between subcutaneous fat thickness in offspring and possible fetal origins of future metabolic disease, particularly given the worsening prevalence of childhood-onset diabetes around the world. While ethnicity was not available in our ultrasound records, residence served as 'proxy' as most northern/rural patients with T2D may have First Nation status; there were no geographic differences between cases and controls which attests to the comparability of genetic/ethnic factors between groups in our study [18]. This observation is in keeping with the fact that patients from rural/northern areas would have had their midpregnancy ultrasound performed in (or near) their home community and not in our regional referral unit. As First Nations status, including the HNF1a gene, is associated with increased risk of T2D, future fetal ultrasound research that engages First Nations community partners is still required to specifically address these influences [18, 44, 45].

Research into the developmental origins of health and disease (“DOHaD”) that occurs in adulthood is inherently challenging because of the difficulty in determining with certainty whether chronic diseases in adulthood are solely related to the intrauterine environment as opposed to all the other potential environmental, cultural, and familial exposures incurred throughout the life course [44–47]. Despite these obstacles, the possibility of early identification of offspring at risk of future disease remains important, and there are multiple studies showing a linkage between maternal medical conditions during pregnancy and health outcomes in offspring later in life [9–13, 47–49]. Fetal ultrasound provides a unique opportunity to study the developmental origins of adult-onset disease given that it is immune to the historic confounders that complicate other DOHaD-related investigations, such as sociodemographic factors or other illnesses and exposures throughout childhood and adulthood. Direct measurement of fetal tissue/structures using objective markers and validated ultrasound techniques that demonstrate differences in organogenesis or fetal development may quell any suspicion about the relationship between fetal exposures and long-term health. Similarly, if fetal changes can be seen in utero that support developmental origins of disease, such findings could be extrapolated towards implementation of early imaging in the postnatal period for prognosticating risk of long-term disease or for measuring the success of prevention strategies. Ongoing work is still required between basic scientists and clinical investigators to understand the pathophysiology of why renal volume changes are seen in utero. For example, do smaller fetal kidneys correspond to reduced renal tubule number in utero or mediated by a different aberration of renal development? With advances in obstetrical ultrasound technology including the three-dimensional volumes. Doppler velocimetry of vascular flow, among others, prenatal diagnosis is improving along with an opportunity to inform other aspects of child health. This study reminds us that the implications of diabetes in pregnancy extend beyond pregnancy for mothers and offspring, and at a minimum, this project increases awareness about the potential application for fetal ultrasound in the effort for earlier risk-stratification and prevention strategies for improvement of population health globally.

Our study is unique as one of the few published about the influence of fetal exposure to maternal T2D on renal size and abdominal wall thickness at midpregnancy in offspring, and provides potential insights into the developmental origins of chronic disease. One strength is that this project was conducted at one of only two regional fetal assessment referral units, meaning that cases are representative of the general obstetrical population from which they are sampled. Another strength is that the addition of formal measurements to fetal kidneys and subcutaneous abdominal wall fat can be done without requiring any additional ultrasound images or healthcare costs. By restricting cases to those with a pregestational diagnosis of T2D, we minimize the risk of selection bias by potentially enrolling patients labelled as “gestational diabetes” who instead represent patients with undiagnosed (and untreated) T2D. Because type 1 diabetes represents the minority of pregestational diabetes cases in our region and not all would necessarily have their anatomy scans performed in our fetal assessment unit (versus a general radiology department), we were underpowered to compare the influence of the different types of pregestational diabetes on fetal renal development.As a case-control design, there are some other inherent limitations of this work.

One limitation is that imaging of fetal kidneys in the sagittal plane is not required as part of routine obstetrical ultrasound between 18 and 22 weeks gestation [27, 28]. For this reason, our evaluation of renal size was restricted to width and thickness measurements only and we are unable to comment on renal length or overall volume. We were also limited by the data available within the fetal assessment unit, which does not routinely collect information about potential confounders such as maternal weight/body mass index or lab data (like haemoglobin A1Cs), or smoking status, and had to use surrogate markers for these covariates. We were limited to data available up to the time of the midpregnancy ultrasound and recorded in our ultrasound charts, and did not have information about late pregnancy complications. However, another study from our group showed no relationship between fetal abdominal wall thickness and the risk of cesarean section or shoulder dystocia [26]. Further research is needed to evaluate the influence of glycemic control, other maternal exposures (including gestational versus type 1 diabetes), ethnicity/HNF1*α* genotype, and preeclampsia on fetal renal development and fat metabolism. Studies about perinatal complications and long-term health conditions are also required to better understand the correlation between fetal renal size (and other ultrasound markers) and future risk of chronic renal disease or other metabolic conditions in offspring exposed to maternal T2D in utero.

## 5. Conclusions

Using standardly obtained images from routine obstetrical ultrasound, we found evidence that fetuses exposed to T2D have significantly smaller kidneys and increased subcutaneous abdominal wall fat thickness compared to healthy controls by midpregnancy, but no difference in estimated fetal weight. Given the known intergenerational effects of T2D and decreased renal tubule numbers in children with youth-onset T2D, these findings may represent evidence of early renal insult which could contribute to an increased risk of future diabetes or diabetic nephropathy amongst fetuses exposed. Further studies are needed to evaluate the utility of fetal ultrasound in predicting long-term health outcomes for offspring at the highest risk for developing chronic renal disease and/or diabetes in the future, and to consider potential prevention strategies for improving health outcomes overall.

## Data Availability

Data are available upon reasonable request.
